# Red Cell Distribution Width and Patient Outcome in Cardiovascular Disease: A ‘’Real-World’’ Analysis

**DOI:** 10.3390/jcdd8100120

**Published:** 2021-09-26

**Authors:** Marisa Talarico, Marcella Manicardi, Marco Vitolo, Vincenzo Livio Malavasi, Anna Chiara Valenti, Daria Sgreccia, Rosario Rossi, Giuseppe Boriani

**Affiliations:** 1 Department of Biomedical, Cardiology Division, Metabolic and Neural Sciences, University of Modena and Reggio Emilia, Policlinico di Modena, Via Del Pozzo n.71, 41124 Modena, Italy; marisa.talarico.2704@gmail.com (M.T.); marcella.manicardi@gmail.com (M.M.); marco.vitolo@unimore.it (M.V.); nanni.malavasi@gmail.com (V.L.M.); annachiaravalenti@gmail.com (A.C.V.); daria.sgreccia@gmail.com (D.S.); rosario.rossi@unimore.it (R.R.); 2 Clinical and Experimental Medicine PhD Program, University of Modena and Reggio Emilia, Via Del Pozzo n.71, 41124 Modena, Italy

**Keywords:** red cell distribution width, RDW, outcome, cardiovascular disease, stroke, biomarkers

## Abstract

Red cell distribution width (RDW) has been shown to predict adverse outcomes in specific scenarios. We aimed to assess the association between RDW and all-cause death and a clinically relevant composite endpoint in a population with various clinical manifestations of cardiovascular diseases. We retrospectively analyzed 700 patients (median age 72.7 years [interquartile range, IQR, 62.6–80]) admitted to the Cardiology ward between January and November 2016. Patients were divided into tertiles according to baseline RDW values. After a median follow-up of 3.78 years (IQR 3.38–4.03), 153 (21.9%) patients died and 247 (35.3%) developed a composite endpoint (all-cause death, acute coronary syndromes, transient ischemic attack/stroke, and/or thromboembolic events). With multivariate Cox regression analysis, the highest RDW tertile was independently associated with an increased risk of all-cause death (adjusted hazard ratio [HR] 2.73, 95% confidence interval [CI] 1.63–4.56) and of the composite endpoint (adjusted HR *2.23,* 95% CI 1.53–3.24). RDW showed a good predictive ability for all-cause death (C-statistics: 0.741, 95% CI 0.694–0.788). In a real-world cohort of patients, we found that higher RDW values were independently associated with an increased risk of all-cause death and clinical adverse cardiovascular events thus proposing RDW as a prognostic marker in cardiovascular patients.

## 1. Introduction

Red cell distribution width (RDW) is a simple and easily available measure of the variation in red blood cells (RBC) size and is routinely reported as a component of the complete blood count. Reference range typically spans between 12–15% for RDW-CV (RDW reported as a coefficient of variation) [1]. Elevated RDW has been shown to predict adverse outcomes in selected cohorts of patients with specific cardiovascular diseases [2] such as acute coronary syndromes [3,4,5,6], heart failure [7], and atrial fibrillation [8,9,10]. The mechanism underlying this phenomenon is not entirely understood. We aimed to evaluate if RDW is an independent predictor of all-cause death and a clinically relevant composite endpoint in an unselected “real world” population of patients with different cardiovascular pathologies.

## 2. Materials and Methods

### 2.1. Patients’ Selection

From 1 January 2016 to 27 November 2016, we retrospectively reviewed 700 consecutive patients admitted to Policlinico di Modena, Cardiology Department, of them, 527 (75.3%) were non-elective patients. Diagnosis at discharge, derived from international classification of diseases (ICD-9) codes, were grouped as follows: chronic coronary syndromes, acute coronary syndromes (ACS), acute heart failure, moderate to severe valvular stenosis/regurgitation, pulmonary embolism, brady- or tachyarrhythmias requiring medical therapy. Patients were excluded if they had missing basal values of RDW, they were <18 years old, and if no follow-up data were available. All data were collected from Hospital Information System ADT^®^ software (Dedalus Healthcare Systems version 03.01.17) and follow-up data were updated based on ISTAT (Italian National Institute of Statistics) death notifications in which the status of all Italian citizens is complete and constantly updated. At enrolment, demographic, clinical, laboratory, and echocardiographic data were collected. Baseline laboratory testing were derived from the first complete blood sample and included: hemoglobin (Hb) concentrations, RBC count, mean corpuscular volume (MCV), mean corpuscular hemoglobin (MCH), mean corpuscular hemoglobin concentration (MCHC), RDW-CV, platelets (PLT) count, white blood cell (WBC) and subtypes count. Basal creatinine concentrations and glomerular filtration rate (GFR), individually calculated according to Chronic Kidney Disease Epidemiology Collaboration (CKD-EPI) formula, were also recorded. We considered reduced GFR as a value < 60 mL/min according to CKD-EPI equation, severe anemia as hemoglobin (HB) levels ≤ 10 g/dL, and reduced left ventricular ejection fraction (LVEF) as a value ≤ 40%, assessed by Biplane method. The study design protocol was approved by the local Ethical Committee (EC).

### 2.2. Study Outcomes

For the purpose of this analysis, the primary outcome was all-cause death. Secondary outcome was the composite endpoint of all-cause death, transient ischemic attack (TIA)/stroke, thromboembolic events, and ACS.

### 2.3. Statistical Analysis

Continuous variables were reported as the median and interquartile range (IQR). Among groups, the comparison was made using a non-parametric test (Kruskal–Wallis). Categorical variables were reported as counts and percentages. Among groups, a comparison was made using Pearson Chi-square or Fisher’s exact test when appropriate. Plots of Kaplan–Meier curves for time to all-cause death and composite endpoint according to RDW tertiles were performed; groups were compared using the log-rank test. Multivariable proportional hazard models were used to estimate the association between RDW tertiles, all-cause death, and the composite endpoint, the lowest tertile was used as a comparator group. We created three multivariable models: Model 1 was adjusted for age and reduced LVEF, Model 2 was adjusted for the same variables of Model 1 plus reduced GFR calculated with CKD-EPI (<60 mL/min) and Model 3 was adjusted for the same variables of Model 2 plus hemoglobin levels and red blood cells count (RBC). Results were expressed as Hazard Ratio (HR, 95% confidence interval CI) and the corresponding *p*-value. Receiver operator curves to analyze RDW predictive ability for all-cause death and composite endpoint were analyzed. For all the analyses, the level of statistical significance was set at a probability value of *p* < 0.05. Statistical analyses were performed using SPSS version 26 software.

## 3. Results

A total of 700 patients were included, the median age was 72.7 years (IQR 62.6–80), 434 were males (62%). The whole population was divided according to baselines RDW values into tertiles as follows: 211 (30.1%) in the lowest (≤13.1 cv%), 254 (36.1%) in the intermediate (13.2–14 cv%), 235 (33.5%) in the highest (≥14.1 cv%). Clinical characteristics according to RDW tertiles are shown in Table 1.

Patients in the highest RDW tertile were older and had a higher prevalence of reduced LVEF and reduced GFR. Acute coronary syndromes were the most prevalent cause of hospital admission among all patients, in the highest RDW tertile heart failure was the second cause of hospital admission (24.4%). Hemoglobin levels, MCV, MCH, and MCHC were progressively lower to increasing tertiles (Table 1).

After a median follow-up of 3.78 years (IQR 3.38–4.03), 153 (21.9%) patients died and 247 (35.3%) developed the composite endpoint (Table 2).

Rate of all-cause death was significantly higher in the highest RDW tertile (42.1% vs. 10% and 13% in the lowest and intermediate tertile respectively, *p* < 0.001). The composite endpoint occurred in 107 (59.8%) patients in the highest RDW tertile vs. 66 (31.4%) and 74 (23.8%) in intermediate and lowest tertile, respectively (*p* < 0.001).

**Figure 1:**
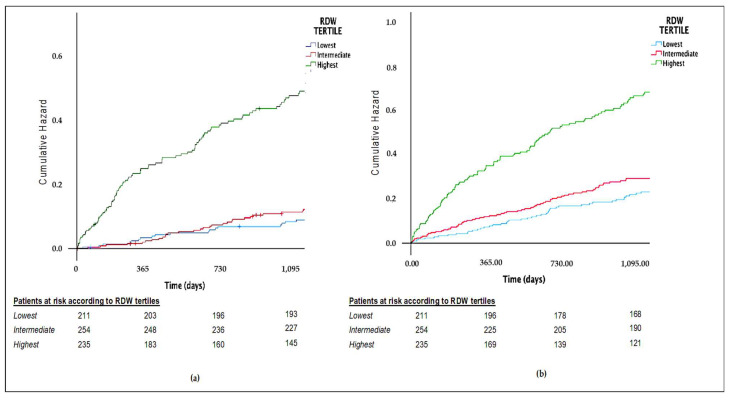
(**a**) Cumulative hazard for all-cause death. (**b**) Cumulative hazard for the composite endpoint.

Kaplan–Meier curves showed a significantly lower cumulative survival probability for both all-cause death and the composite endpoint in the highest tertile (Figure 1a,b).

With the multivariable Cox regression analysis (Table 3 and Table 4), after multiple adjustments, the highest RDW tertile was independently associated with an increased risk of all-cause death (Model 1, HR 3.36, 95% CI 2.06–5.46; Model 2, HR 2.7, 95% CI 1.64–4.44; Model 3, HR 2.73, 95% CI 1.63–4.56). The highest RDW tertile was found to be independently associated with an increased risk of the composite endpoint, even after multiple adjustments (Model 1, HR 2.45, 95% CI 1.73–3.47; Model 2, HR 2.13, 95% CI 1.49–3.01; Model 3, HR 2.23, 95% CI 1.53–3.24).

**Figure 2:**
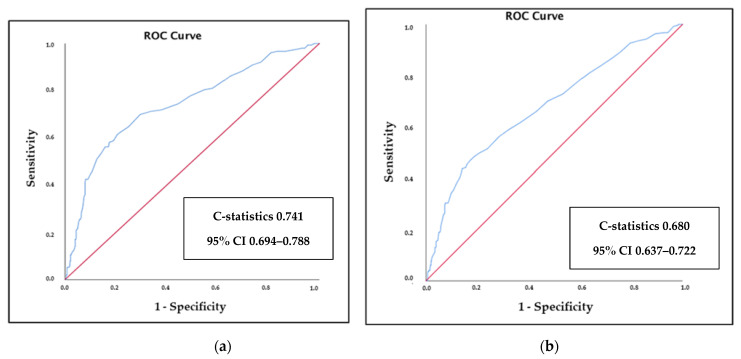
(**a**) ROC curve for mortality according to RDW. (**b**) ROC curve for composite endpoint according to RDW.

ROC curves showed that RDW had an acceptable predictive ability for all-cause death (C-statistics: 0.741, 95% CI 0.694–0.788) and a modest predictive ability for the prediction of the composite endpoint (C-statistics: 0.680, 95% CI 0.637–0.722) (Figure 2a,b).

## 4. Discussion

The present study from a real-world cohort of patients with cardiovascular diseases found that subjects presenting with higher RDW values have a worse clinical profile (in terms of the prevalence of chronic kidney disease, reduced left ventricular ejection fraction, advanced age).

These findings may be interpreted taking into account that, according to literature, and besides its usefulness in the differential diagnosis of anemias, RDW reflects abnormalities in erythropoiesis related to aging, oxidative stress, and systemic inflammatory state [11].

In our cohort, ACS were the most common cause of hospital admission in all RDW tertiles, while the prevalence of acute heart failure was higher for patients in the highest RDW tertile compared to intermediate and lowest tertiles.

Several studies have investigated the prognostic role of RDW both in acute coronary syndromes and heart failure. In a population of 1654 patients admitted for ACS, Wang et al. highlighted that increased RDW values were independent predictors of cardiac mortality (odds ratio [OR] 2.1, 95% CI 1.4–3.1) during a one-month follow-up [12]. In a meta-analysis involving 10,410 patients, Abrahan et al. [13] found that a low RDW during an ACS was associated with a lower risk of all-cause death or cardiovascular mortality (RR 0.35, (95% CI 0.30 to 0.40), *p* < 0.00001, I^2^ = 53%) and lower risk of major adverse cardiovascular events (risk ratio (RR) 0.56, (95% CI 0.51 to 0.61), *p* < 0.00001, I^2^ = 91%). Interestingly, in a recent metanalysis involving twelve studies, high RDW predicted all-cause mortality among 17,113 patients with coronary artery disease undergoing percutaneous coronary intervention, with a stronger predictive effect in the non-anemic subgroup compared to the anemic one (RR 4.59; 95%CI 3.07 to 6.86 vs. RR 1.77; 95%CI 1.32 to 2.37) [14].

The prognostic role of RDW has been described also among patients with heart failure, in whom higher degrees of anisocytosis increased both all-cause mortality and the risk of adverse events in several studies and meta-analyses [15,16,17]. Liu et al. [18] studied the predictive value of RDW for mortality among patients hospitalized for heart failure, finding that RDW was an independent risk factor for mortality (OR = 2.531, 95% CI 1.371–4.671). As in the setting of ACS, the independent association between RDW values and all-cause mortality was observed both in anemic and non-anemic heart failure patients [19].

Of note, despite the well-known relation between RDW and anemias, the prognostic role of RDW seems to be independent of Hb levels. Our results support this statement, as the relation between higher RDW values and worse outcomes remained significant even after adjusting for Hb levels and RBC count. The strength of RDW, expressed in tertile, as a variable associated with adverse outcomes, was confirmed in all the Cox models that were tested, with the highest RDW tertile found to be associated with all-cause death and the composite endpoint, as shown in Supplementary Tables S1 and S2. In accordance with our findings, a recent metanalysis on 102,689 participants [17] reported a pooled HR of 1.12 (95% CI = 1.09–1.15) for the association of all-cause mortality per 1% increase in RDW and 1.12 (95% CI 1.08–1.17) for major adverse cardiac events (MACEs) per 1% increase in RDW. In addition, a dose-response curve relating RDW increase to its effect on cardiovascular outcome was also reported: for every 1-unit increase in RDW there was an increased risk of occurrence of all-cause mortality (pooled HR = 1.03, 95% CI = 1.02–1.04) and MACEs (pooled HR = 1.04, 95% CI = 1.01–1.06). To adjust the confounding of anemia the ratio RDW to Hb (RDW/Hb) was calculated and an additional meta-analysis to evaluate its prognostic role was performed. For every 1-unit increase in RDW/Hb, pooled HR for all-cause death was 2.03 (95% CI = 1.60–2.57) and 1.58 (95% CI= 1.09–2.29) for MACEs.

Our study was focused on a real-life cohort of unselected patients admitted to a Cardiology ward for various manifestations of cardiovascular diseases, and we highlighted that higher RDW values are independently associated with the risk of all-cause death and of a composite endpoint of clinically relevant events (all-cause death, TIA/stroke, thromboembolic events, acute coronary syndromes) even after adjustments for potential confounders: age, reduced LVEF, chronic kidney disease, Hb and RBC levels.

Many plausible mechanisms have been hypothesized to explain the link between higher RDW values and worse outcomes among cardiovascular diseases. The most attractive of them involve the role of higher RDW in promoting endothelial dysfunction, vascular damage, and changes in the cholesterol content of the RBC membrane, all these mechanisms being involved in the pathogenesis, progression, and instability of atherosclerotic plaque. Furthermore, the decrease in RBC deformability associated with higher RDW values may slow the blood flow through the microcirculation finally triggering hypoxia and ischemic processes [11]. Above all, chronic inflammation and oxidative stress, which impact bone-marrow progenitors contribute to anisocytosis, may also promote adverse cardiac remodeling and favor the development and progression of heart failure [20].

However, the complex interplay between RDW and mortality has not yet been fully characterized.

In the last years, several studies have evaluated the association between the risk of all-cause death and RDW in the general population [21,22]. In a retrospective study on 8175 subjects, Patel et al. [22] found that higher RDW values were strongly associated with an increased risk of death, for every 1% increment in RDW, all-cause mortality risk increased by 22% (HR 1.22, 95% CI 1.15–1.30, *p* < 0.001).

A more recent population-based cohort study on 27,063 patients [23], followed for 19.8 ± 5.5 years, showed that high RDW was significantly associated with all-cause mortality, even after adjustments for confounding factors (HR highest vs. lowest RDW quartile: 1.34, 95% CI 1.24–1.45). The C-statistics for all-cause mortality from a model including age and sex increased when RDW was added to the model (from 0.732 (95% CI 0.727–0.737) to 0.737 (95% CI 0.732–0.742)). In line with previous findings, in our study RDW showed a good predictive ability for all-cause death but only a modest ability in the prediction of the composite endpoint.

The inherited genetic variation associated with RDW was investigated in 116,666 UK Biobank human volunteers [24]. The genetic risk scores analysis found that higher RDW was associated with lower low-density lipoproteins (LDL) levels or systolic pressure, while the proportion of the variance shared between RDW, and coronary heart disease was only 6.6%. Outcome implications of increased RDW were not explained by diagnosed cardiovascular disease, related lipid genetic risks, or an RDW genetic score, suggesting that the predictive value of RDW for a range of negative health outcomes may in part be related to variants influencing fundamental pathways of aging.

Our study adds the clinically valuable information that RDW may be considered a low-cost marker with implications for patient outcome also in the specialized context of patients admitted for various conditions to a cardiology ward. RDW, as derived from baseline laboratory assessment has therefore the potential to integrate clinical prediction of patient outcome, independently on widely used outcome predictors.

Some limitations of our study should be acknowledged. First, our cohort represents a single-center experience, and the study has a relatively limited sample size, thus limiting the generalization of the results. Given the observational nature of the study, the statistical power of the analysis is limited, and the possibility of unmeasured confounders cannot be excluded. The data presented do not imply causality, rather describe an association.

A strength of the study is represented by the extensive and complete follow-up of the study.

## 5. Conclusions

In a retrospective study performed on a “real-world” cohort of cardiovascular patients, RDW is associated with clinical factors indicating a worse profile. Higher RDW values are independently associated with an increased risk of all-cause death and a composite clinically relevant endpoint.

RDW is an easily available and low-cost biomarker that can help clinicians in improving the identification of patients at higher risk of adverse outcomes. Further studies are needed to understand the changes along with the time of RDW.

## Figures and Tables

**Table 1:** Demographic, clinical, and laboratory characteristics of patients enrolled.

		RDW Tertiles			
	Overall Cohort (*n* = 700)	Lowest (≤13.1% cv) (*n* = 211)	Intermediate (13.2%–14% cv) (*n* = 254)	Highest (≥14.1% cv) (*n* = 235)	*p*-Value
**Demographic Characteristics**					
Age	72.7 [62.6–80]	67.7 [56.5–76.7]	72.6 [62.9–79.8]	76.7 [67.5–83]	<0.01
Male sex	434 (62%)	132 (62.6%)	163 (64.2%)	139 (59.1%)	0.510
BMI	26.6 [23.8–29.4]	25.8 [24.1–29.2]	27 [23.6–29.4]	26.8 [23.9–30.1]	0.546
**Clinical Characteristics**					
Left ventricular ejection fraction <40%	115 (16.4%)	24 (11.6%)	30 (11.8%)	61 (26%)	<0.001
GFR CKD-EPI < 60 mL/min	209 (29.9%)	28 (13.3%)	63 (24.8%)	118 (50.2%)	<0.001
Hypertension	471 (67.3%)	132 (62.6%)	172 (67.7%)	167 (71.1%)	0.158
Diabetes	168 (24%)	36 (17.1%)	72 (28.3%)	60 (25.5%)	0.014
Dyslipidemia	353 (50.4%)	121 (57.3%)	126 (49.6%)	106 (45.1%)	0.034
Active smokers	196 (28%)	60 (28.4%)	80 (31.5%)	56 (23.8%)	0.166
Non elective hospital admissions	527 (75.3%)	152 (72%)	183 (72%)	192 (81.7%)	0.020
Diagnosis at discharge					<0.001
Chronic coronary syndromes	93 (13.3%)	33 (15.6%)	43 (16.9%)	17 (7.2%)	
Acute coronary syndromes	289 (41.3%)	107 (50.7%)	105 (41.3%)	77 (32.8%)	
Heart failure	103 (14.7%)	9 (4.3%)	25 (9.8%)	69 (24.4%)	
Valvular diseases and PE	86 (12.3%)	33 (15.6%)	30 (11.8%)	23 (9.8%)	
Arrhythmias	129 (18.4%)	29 (13.7%)	51 (20.1%)	40 (20.9%)	
**Laboratory Parameters**					
HB (g/dL), median [IQR]	13.3 [11.9–14.4]	13.9 [12.9–14.7]	13.5 [12.3–14.6]	12 [10.8–13.4]	<0.001
MCV (fl), median [IQR]	88 [85–91.2]	88.4 [85.9–90.9]	88 [85.7–91.4]	86.9 [81.8–91.5]	0.004
MCH (pg), median [IQR]	29.7 [28.3–30.8]	29.9 [29.2–31]	29.9 [28.9–30.8]	28.6 [26.2–30.3]	<0.001
MCHC (g/dL), median [IQR]	33.6 [32.7–34.3]	34 [33.5–34.5]	33.7 [33–34.4]	32.6 [31.6–33.7]	<0.001
WBC (× 10^3^/mm^3^), median [IQR]	7.4 [6–9.1]	7.2 [5.7–9]	7.3 [6–9.2]	7.5 [6–9.3]	0.430
RBC (× 10^9^/mm^3^), median [IQR]	4.5 [4.1–4.9]	4.5 [4.2–4.9]	4.5 [4.1–4.9]	4.3 [3.9–4.8]	<0.001
PLT (× 10^3^/mmc), median [IQR]	203 [169–244]	206 [177–239]	199 [165.5–235]	204 [163–257]	0.469
HCT (%), median [IQR]	39.5 [36–42.9]	40.6 [37.8–43.4]	40 [36.7–43.1]	37.2 [34.1–41.2]	<0.001
Neutrophils (× 10 ^3^/mm^3^), median [IQR]	4.7 [3.5–6.1]	4.4 [3.4–5.7]	4.7 [3.4–6]	4.9 [3.8–6.6]	0.007
Lymphocyte (× 10^3^/mm^3^), median [IQR]	1.8 [1.3–2.3]	1.9 [1.4–2.4]	1.8 [1.4–2.3]	1.6 [1.2–2.3]	<0.001
RDW (cv%), median [IQR]	13.7 [13–14.6]	12.7 [12.4–13]	13.6 [13.4–13.8]	15.2 [14.6–16.4]	<0.001
Creatinine (mg/dL), median [IQR]	0.9 [0.8–1.2]	0.9 [0.7–1]	0.9 [0.8–1.1]	1 [0.9–1.5]	<0.001
GFR CKD-EPI (mL/min BSA), median [IQR]	75.5 [52.9–89.4]	85.3 [73.5–96.2]	77.4 [59–89.8]	58.9 [37.9–77.1]	<0.001

BMI, body mass index; EF, ejection fraction; GFR CKD-EPI, glomerular filtration rate according to Chronic Kidney Disease Epidemiology Collaboration; PE, pulmonary embolism; HB, hemoglobin; MCV, mean corpuscular volume; MCH, mean corpuscular hemoglobin; MCHC, mean corpuscular hemoglobin concentration; WBC, white blood cells; RBC, red blood cells; PLT, platelets; HCT, hematocrit; RDW, red cell distribution width.

**Table 2:** Major adverse clinical events during the follow-up.

		RDW Tertiles			
	Overall Cohort (*n* = 700)	Lowest (≤13.1% cv) (*n* = 211)	Intermediate (13.2%–14% cv) (*n* = 254)	Highest (≥14.1% cv) (*n* = 235)	*p*-Value
All cause deaths	153 (21.9%)	21 (10%)	33 (13%)	99 (42.1%)	<0.001
Acute coronary syndromes	104 (14.9%)	24 (11.4%)	40 (15.7%)	40 (17%)	0.217
TIA/stroke	26 (3.7%)	5 (2.4%)	9 (3.5%)	12 (5.1%)	0.307
Thromboembolic events	3 (0.4%)	1 (0.5%)	1 (0.4%)	1 (0.4%)	0.991
Composite end-point	247 (35.3%)	74 (23.8%)	66 (31.4%)	107 (59.8%)	<0.001

TIA, transient ischemic attack; composite endpoint (all cause death, acute coronary syndromes, thromboembolic events, TIA/stroke).

**Table 3:** Unadjusted and adjusted Cox regression analysis for all-cause death.

			Adjusted Analysis					
RDW Tertile	Unadjusted Analysis		Model 1 *		Model 2 **		Model 3 ***	
	HR	95% CI, *p*-Value	HR	95% CI, *p*-Value	HR	95% CI, *p*-Value	HR	95% CI, *p*-Value
Lowest *(ref.)*	-	-	-	-	-	-	-	-
Intermediate	1.34	0.77–2.31, *p* = 0.298	1.02	0.59–1.77, *p* = 0.942	0.89	0.52–1.56, *p* = 0.699	0.92	0.52–1.60, *p* = 0.763
Highest	5.42	3.39–8.7, *p* < 0.001	3.36	2.06–5.46, *p* < 0.001	2.70	1.64–4.44, *p* < 0.001	2.73	1.63–4.56, *p* < 0.001

Legend: * = adjusted for age and reduced LVEF (< 40%); ** adjusted for the same variables of model 1 plus reduced GFR calculated with CKDEPI (<60 mL/min); *** adjusted for the same variables of model 2 plus hemoglobin levels and RBC.

**Table 4:** Unadjusted and adjusted Cox regression analysis for the composite outcome.

			Adjusted Analysis					
RDW Tertile	Unadjusted Analysis		Model 1 *		Model 2 **		Model 3 ***	
	HR	95% CI, *p*-Value	HR	95% CI, *p*-Value	HR	95% CI, *p*-Value	HR	95% CI, *p*-Value
Lowest (*ref.*)	-	-	-	-	-	-	-	-
Intermediate	1.35	0.94–1.95, *p* = 0.106	1.15	0.80–1.67, *p* = 0.439	1.09	0.75–1.59, *p* = 0.638	1.13	0.78–1.63, *p* = 0.532
Highest	3.20	2.29–4.48, *p* < 0.001	2.45	1.73–3.47, *p* < 0.001	2.13	1.49–3.01, *p* < 0.001	2.23	1.53–3.24, *p* < 0.001

Legend: * = adjusted for age and reduced LVEF (<40%); ** adjusted for the same variables of model 1 plus reduced GFR calculated with CKDEPI (<60 mL/min); *** adjusted for the same variables of model 2 plus hemoglobin levels and RBC.

## Data Availability

The data presents in this study are available on request from the corresponding author. The data are not publicly available due to privacy restrictions.

## Supplementary Materials

The followings are available online at https://www.mdpi.com/article/10.3390/jcdd8100120/s1, Table S1: adjusted Cox regression analysis for all cause death. (A) Model 1, adjusted for age and reduced LVEF (<40%) (B) Model 2, adjusted for age, reduced LVEF and reduced eGFR (<60 mL/min calculated with CKDEPI) (C) Model 3, adjusted for age, reduced LVEF, reduced eGFR, Hb and RBC. Table S2: adjusted Cox regression analysis for the composite outcome. (A) Model 1, adjusted for age and reduced LVEF (<40%) (B) Model 2, adjusted for age, reduced LVEF and reduced eGFR (<60 mL/min calculated with CKDEPI) (C) Model 3, adjusted for age, reduced LVEF, reduced eGFR, Hb and RBC.
