# Supplementary material for: Red Cell Distribution Width and Patient Outcome in Cardiovascular Disease: A ‘’Real-World’’ Analysis
Source: J Cardiovasc Dev Dis. 2021 Sep 26;8(10):120. doi: 10.3390/jcdd8100120 (PMC8539630; doi:10.3390/jcdd8100120)
Supplement: Supplementary file 1 [file jcdd-08-00120-s001.zip › jcdd-1326103-supplementary.pdf]

Supplementary Table S1 adjusted Cox regression analysis for all cause death. A) Model 1, adjusted for age and reduced LVEF (<40%) B) Model 2, adjusted for age, reduced LVEF and reduced eGFR (< 60 mL/min calculated with CKDEPI) C) Model 3, adjusted for age, reduced LVEF, reduced eGFR, Hb and RBC.

A) Model 1

	HR	95,0% CI		p value
		Lower	Upper	
Age	1.07	1.05	1.09	p< 0.001
Reduced LVEF	1.22	0.83	1.79	p = 0.311
Lowest RDW tertile *(ref)*				
Intermediate RDW tertile	1.02	0.59	1.77	p = 0.942
Highest RDW tertile	3.36	2.06	5.46	p < 0.001

B) Model 2

	HR	95,0% CI		p value
		Lower	Upper	
Age	1.05	1.03	1.07	p < 0.001
Reduced LVEF	1.12	0.77	1.65	p = 0.548
Lowest RDW tertile *(ref)*				
Intermediate RDW tertile	0.89	0.52	1.56	p = 0.699
Highest RDW tertile	2.7	1.64	4.44	p < 0.001
Reduced eGFR	2.49	1.72	3.58	p < 0.001

C) Model 3

	HR	95,0% CI		p value
		Lower	Upper	
Age	1.05	1.03	1.07	p < 0.001
Reduced LVEF	1.11	0.75	1.6	0.604
Lowest RDW tertile *(ref)*				
Intermediate RDW tertile	0.92	0.52	1.6	p = 0.763
Highest RDW tertile	2.73	1.63	4.56	p < 0.001
Reduced eGFR	2.11	1.44	3.09	p < 0.001
RBC	0.65	0.41	1.00	p = 0.052
Hb	1.02	0.88	1.19	p = 0.792

Legend: reduced LVEF (<40%); reduced GFR calculated with CKDEPI (< 60 mL/min); RBC, red blood cells; Hb, haemoglobin.

Supplementary Table S2 adjusted Cox regression analysis for the composite outcome. A) Model 1, adjusted for age and reduced LVEF (<40%) B) Model 2, adjusted for age, reduced LVEF and reduced eGFR (< 60 mL/min calculated with CKDEPI) C) Model 3, adjusted for age, reduced LVEF, reduced eGFR, Hb and RBC.

A) Model 1

	HR	95,0% CI		p value
		Lower	Upper	
Age	1.03	1.02	1.05	p < 0.001
LVEF	0.98	0.71	1.36	p = 0.92
Lowest RDW tertile *(ref)*				
Intermediate RDW tertile	1.15	0.80	1.67	p = 0.439
Highest RDW tertile	2.45	1.73	3.47	p < 0.001

B) Model 2

	HR	95,0% CI		p value
		Lower	Upper	
Age	1.02	1.01	1.04	p < 0.001
LVEF	0.942	0.68	1.31	p = 0.720
Lowest RDW tertile *(ref)*				
Intermediate RDW tertile	1.09	0.75	1.59	p = 0.638
Highest RDW tertile	2.13	1.49	3.01	p < 0.001
eGFR	1.86	1.40	2.48	p < 0.001

C) Model 3

	HR	95,0% CI		p value
		Lower	Upper	
Age	1.02	1.01	1.03	p = 0.001
LVEF	0.99	0.67	1.29	p = 0.657
Lowest RDW tertile *(ref)*				
Intermediate RDW tertile	1.13	0.78	1.63	p = 0.532
Highest RDW tertile	2.23	1.53	3.24	p < 0.001
eGFR	1.68	1.24	2.26	p = 0.001
RBC	0.71	0.51	0.98	p = 0.039
Hb	1.04	0.93	1.16	p = 0.503

Legend: reduced LVEF (<40%); reduced eGFR calculated with CKDEPI (< 60 mL/min); RBC, red blood cells; Hb, haemoglobin.
